# The Role of Fluorine Substituents on the Physical Properties of 4-Pentyl-4″-propyl-1,1′:4′,1″-terphenyl Liquid Crystals

**DOI:** 10.1021/acs.jpcb.5c00140

**Published:** 2025-03-18

**Authors:** Anna Drzewicz, Przemysław Kula, Ewa Juszyńska-Gałązka

**Affiliations:** a The Henryk Niewodniczański Institute of Nuclear Physics Polish Academy of Sciences, PL-31342 Krakow, Poland; b Institute of Chemistry, Military University of Technology, PL-00908 Warszawa, Poland; c Research Center for Thermal and Entropic Science, Graduate School of Science, Osaka University, 560-0043 Osaka, Japan

## Abstract

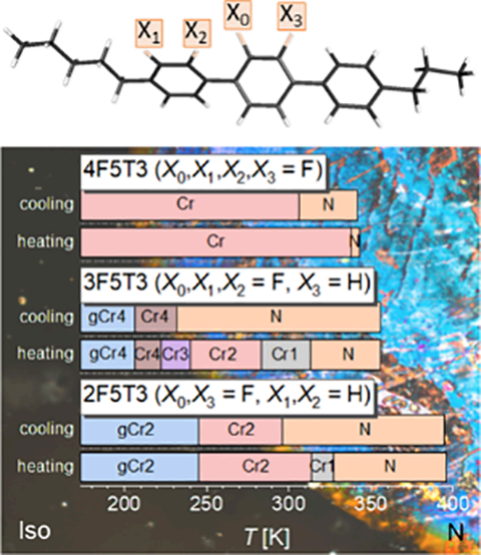

The phase behavior of three liquid crystal derivatives of 4-pentyl-4″-propyl-1,1′:4′,1″-terphenyls differing in number of fluorine atoms in the mesogenic core has been described. Calorimetric and microscopic studies show that the fluoro-substitution of the rigid core affects whether a given liquid crystal undergoes crystallization or a glass transition during cooling. Compounds with the highest number of fluorine atoms crystallize, while other derivatives vitrify from the conformationally disordered crystal phase. Calorimetric studies show different mechanisms of cold crystallization processes. Dielectric measurements uncover the complex relaxation processes present in the nematic and crystal phases and glassy states. DFT calculations allow for the determination of which stochastic movements are responsible for specific relaxation processes. Infrared absorption spectra show that the C–H bending vibrations are a sensitive probe of changes to the local surrounding of a molecule related to the thermodynamic states.

## 1 Introduction

**Figure 1:**
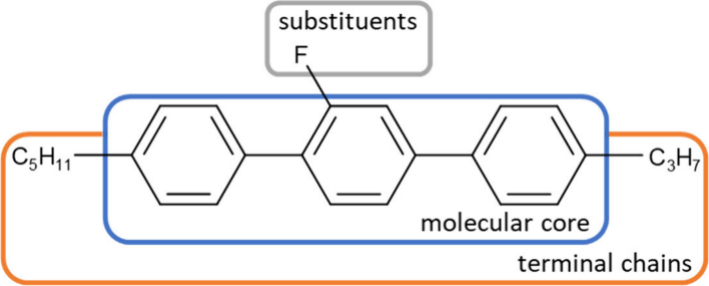
Scheme of a typical mesogenic structure.

Although liquid crystals are primarily associated with the display industry, over the past several years, alternative applications of liquid crystals as promising smart soft materials have been demonstrated. For example, light-driven chiral LCs have been used as biosensors for detecting proteins, nucleic acids, and viruses,^1^ luminescent materials with circularly polarized light for quantum communication,^2^ or discotic liquid crystal porphyrins with partial chain perfluorination for nanoproduction of molecular devices.^3^ However, to fully understand the potential applications of liquid crystals, it is essential to first characterize them, ideally focusing on the impact of individual structural elements on the mesomorphic properties of the compound. Liquid crystal molecules are typically composed of the following elements: a rigid core formed by a system of rings, connected or not by linking groups, and terminal chains, groups, or substituents, as in Figure 1. The complex molecular structure is one of the factors influencing the phase behavior of liquid crystals. For instance, the type and position of lateral substitutions on the benzene rings of the molecular core significantly lower the phase transition temperatures compared to unsubstituted materials.^4^ The linking bridge type and the length of alkyl chains affects the phase situation of fluorinated terphenyls.^5,6^ Terphenyls with a shorter alkyl chain (up to the pentyl group) are typical nematogens, and an increase in the alkyl chain length leads to the occurrence of smectic phases. *para*-Terphenyls attracted considerable attention in structural studies and conformational analyses due to their conjugated π-electronic structure generated by delocalized electrons in the terphenyl part.^7,8^

Due to the unique characteristics of fluorine, including its electronic, simulation, hindering, and permeation effects, the incorporation of fluorine atoms into liquid crystals significantly alters their properties. In comparison to non-fluorinated liquid crystals, fluorinated counterparts exhibit several advantages: optimal dielectric anisotropy, enhanced resistivity, improved voltage retention, reduced viscosity, better performance at low temperatures, superior compatibility, and reduced temperature sensitivity. Especially fluorinated terphenyls are considered promising components in ferroelectric mixtures due to their low viscosity, low conductivity, and high chemical and thermal stability.^9^ Among terphenyls, one can find nematics (N) with negative dielectric anisotropy,^10^ medium birefringence nematics,^11^ smectics (Sm),^12^ cholesterics (Ch),^13^ ferroelectrics (FLCs),^14^ antiferroelectrics (AFLCs),^15^ and twist-bend nematics (NTB).^16^ Terphenyl derivatives find applications in many fields, including medicine (e.g., as antitumor agents^17^) or advanced technology (e.g., in organic light-emitting diodes (OLEDs),^18^ in bulk heterojunction solar cells,^19^ in organic thin-film transistors (OTFTs),^20^ in laser dyes,^21^ in helical polymers,^22^ and in molecular electronic devices^23^).

**Figure 2:**
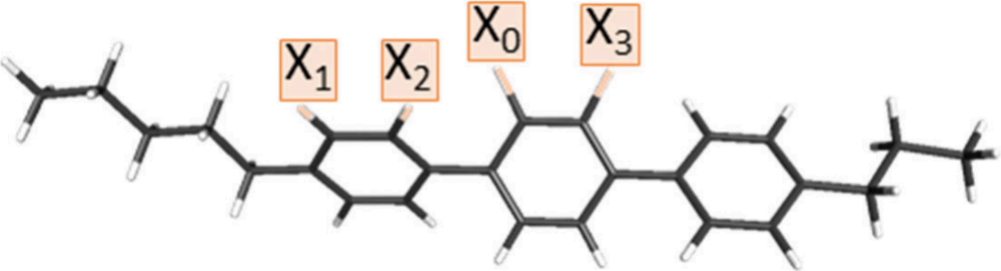
General formula of the investigated *n*F5T3 compounds, where *n* indicates the number of fluorine atoms (*n* = 2–4), X_0_ is a fluorine atom, and X_1_, X_2_, and X_3_ are fluorine (F) or hydrogen (H) atoms. Legend: X_1_, X_2_ = H, X_0_, X_3_ = F, *n* = 2, for 2F5T3; X_3_ = H, X_0_, X_1_, X_2_ = F, *n* = 3 for 3F5T3; X_0_, X_1_, X_2_, X_3_ = F and *n* = 4 for 4F5T3.

We report here the results of the detailed investigation of three fluoro-substituted terphenyl derivatives: 2′,3′-difluoro-4-pentyl-4″-propyl-1,1′:4′,1″-terphenyl (abbreviated as 2F5T3), 2,3,2′-trifluoro-4-pentyl-4″-propyl-1,1′:4′,1″-terphenyl (abbreviated as 3F5T3), and 2,3,2′,3′-tetrafluoro-4-pentyl-4″-propyl-1,1′:4′,1″-terphenyl (abbreviated as 4F5T3). The general formulas of these compounds are presented in Figure 2. All molecules have the same terminal alkyl chains and a fluorine atom in the X_0_ position in the molecular core, while they differ in the number and placement of fluorine atoms in the remaining X_1_, X_2_, and X_3_ positions in the terphenyl mesogenic core. Our primary objective is to examine how the fluoro-substitution of the rigid core influences the self-assembly behavior and the relaxation dynamics of the *n*F5T3 (*n* is the number of fluorine atoms in the molecular core, *n* = 2–4) compounds. These studies on the physicochemical properties of fluoro-substituted terphenyl derivatives align with current research focused on dynamic changes occurring in the thermodynamic states of terphenyl-based liquid crystals. The impact of terminal substituents,^24^ fluorination of alkyl chains,^25^ length of terminal aliphatic chains,^10^ chiral terminal group,^26^ and lateral halogens^27^ on liquid crystalline properties for terphenyl-based molecules have been recently described. One of the missing elements is the influence of the number and position of fluorine atoms in the molecular core on the mesomorphic properties of terphenyls, which is the subject of this work. Determining how individual elements of the molecular structure of a given compound family affect their self-assembly behavior could help in the design of liquid crystals with desired properties for specific applications.

## 2 Experimental Section

The *n*F5T3 compounds were synthesized at the Institute of Chemistry, Military University of Technology, Warsaw, Poland. For one of the considered compounds (i.e., for 2F5T3), the general synthetic method and chosen mesomorphic properties are described in refs (12 and 28). The purity of the samples was determined to be over 99.0% using the ASTM E928 Standard Test Method for Determining Purity by Differential Scanning Calorimetry.

Differential Scanning Calorimetry (DSC) thermograms were registered using a TA DSC 2500 calorimeter. The indium and sapphire standards were used for calibration. The samples with the masses ca. 5 mg were cooled and heated at several rates (±2, ±5, ±8, ±10, ±15, ±20, ±25, and ±30 °C min^–1^). The modulated-temperature (MT-DSC) regime measurements were performed for 3F5T3 at a heating rate of 3 °C min^–1^ with a temperature modulation amplitude of 1 °C and a modulation period of 60 s. MT-DSC provides more information about the thermodynamic and kinetic details of the phase transitions.^29^ The total heat flow corresponds to what is typically measured by traditional DSC and is made up of two components: the reversing heat flow, which includes heat capacity, and the nonreversing heat flow, which includes the kinetic component.

Polarizing Optical Microscopy (POM) textures were observed using a Leica DM2700P microscope with the crossed polarizers equipped with a Linkam LNP96-S heating/cooling stage and a Linkam T96-S temperature controller. The samples were placed on covered glass plates without aligning layers in the isotropic liquid phase and were cooled and heated at a rate of 5 °C min^–1^. The data analysis was done in the TOApy program.^30^

Broadband Dielectric Spectroscopy (BDS) measurements were performed using a high-resolution Novocontrol Alpha Analyzer with a Novocontrol temperature controller, in the frequency range of 0.1 Hz to 10 MHz. The samples were placed between two gold electrodes with an active area of 10 mm, without aligning layers and with poly(tetrafluoroethylene) spacers with a thickness of 74 μm. Dielectric spectra were collected upon heating at a rate of 2 °C min^–1^ after fast cooling.

Fourier Transformed Infrared (FTIR) spectra for 3F5T3 were recorded using a Bio-Rad Digilab FTS 3000 Excalibur spectrometer in the wavenumber range of 4000–500 cm^–1^, with a resolution of 0.4 cm^–1^ and taking 64 scans. The samples in the form of thin films were placed between two zinc selenide window discs. Infrared spectra were collected upon cooling at a rate of 2 °C min^–1^. The Perturbation–Correlation Moving Window Two-Dimensional (PCMW2D) and Two-Dimensional Correlation (2D-COS) analyses of hyperspectral data were performed by means of the 2D-SHIGE program.^31^

All measurements were carried out under a nitrogen atmosphere in the temperature range from −100 °C to the transition temperature to the isotropic liquid phase.

Quantum chemical DFT calculations were determined for the isolated molecules in several conformations using TURBOMOLE v7.3 at the Academic Computer Centre CYFRONET (Krakow), with the basis set of def2-TZVPPD for all atoms, the B3-LYP functional, and the D4 dispersion corrections.

## 3 Results and Discussion

### 3.1 Thermal Phase Behavior of *n*F5T3

**Figure 3:**
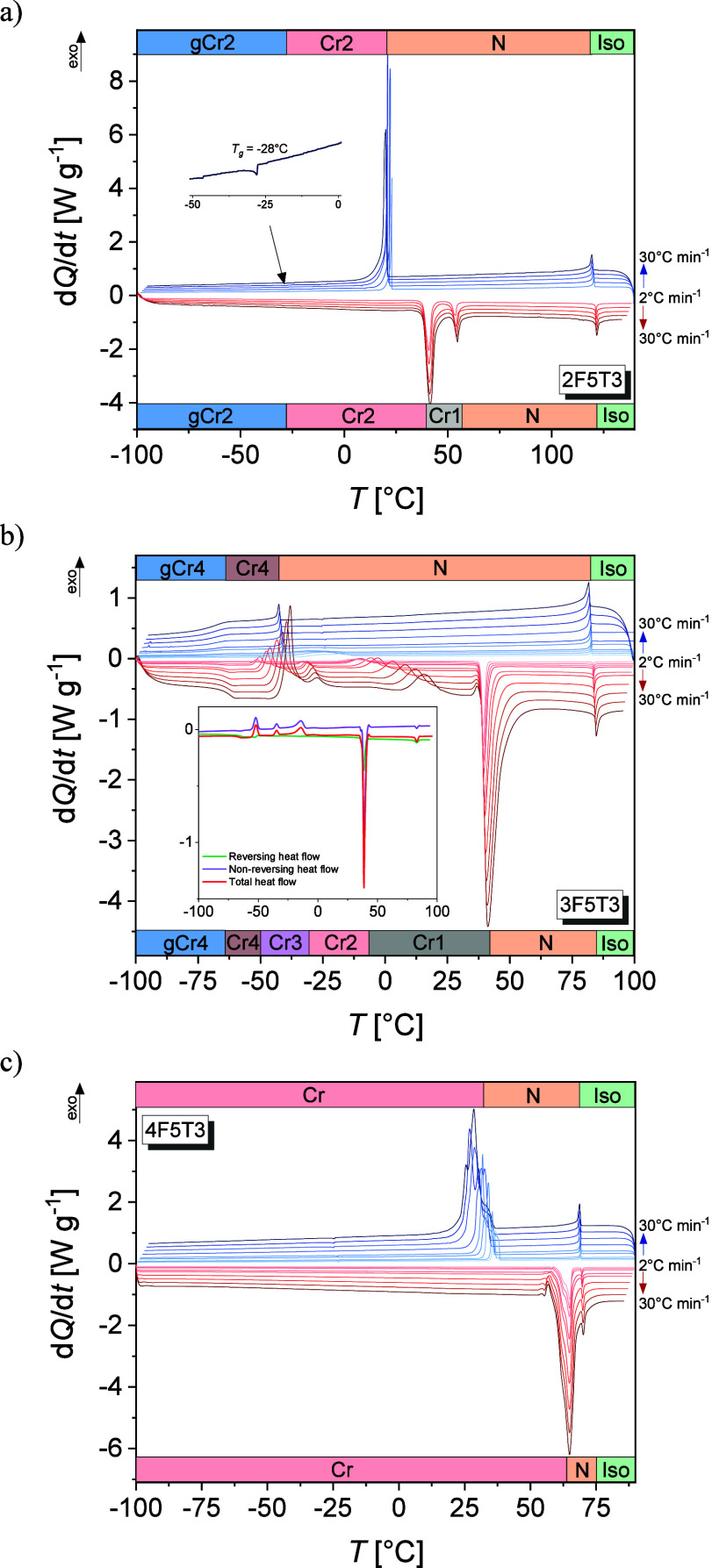
DSC thermograms of the 2F5T3 (a), 3F5T3 (b), and 4F5T3 (c) collected upon cooling (blue) and heating (red). Inset in panel b presents the MT-DSC thermograms for a heating rate of 3 °C min^–1^ with a modulation amplitude of 1 °C for a period of 60 s.

**Figure 4:**
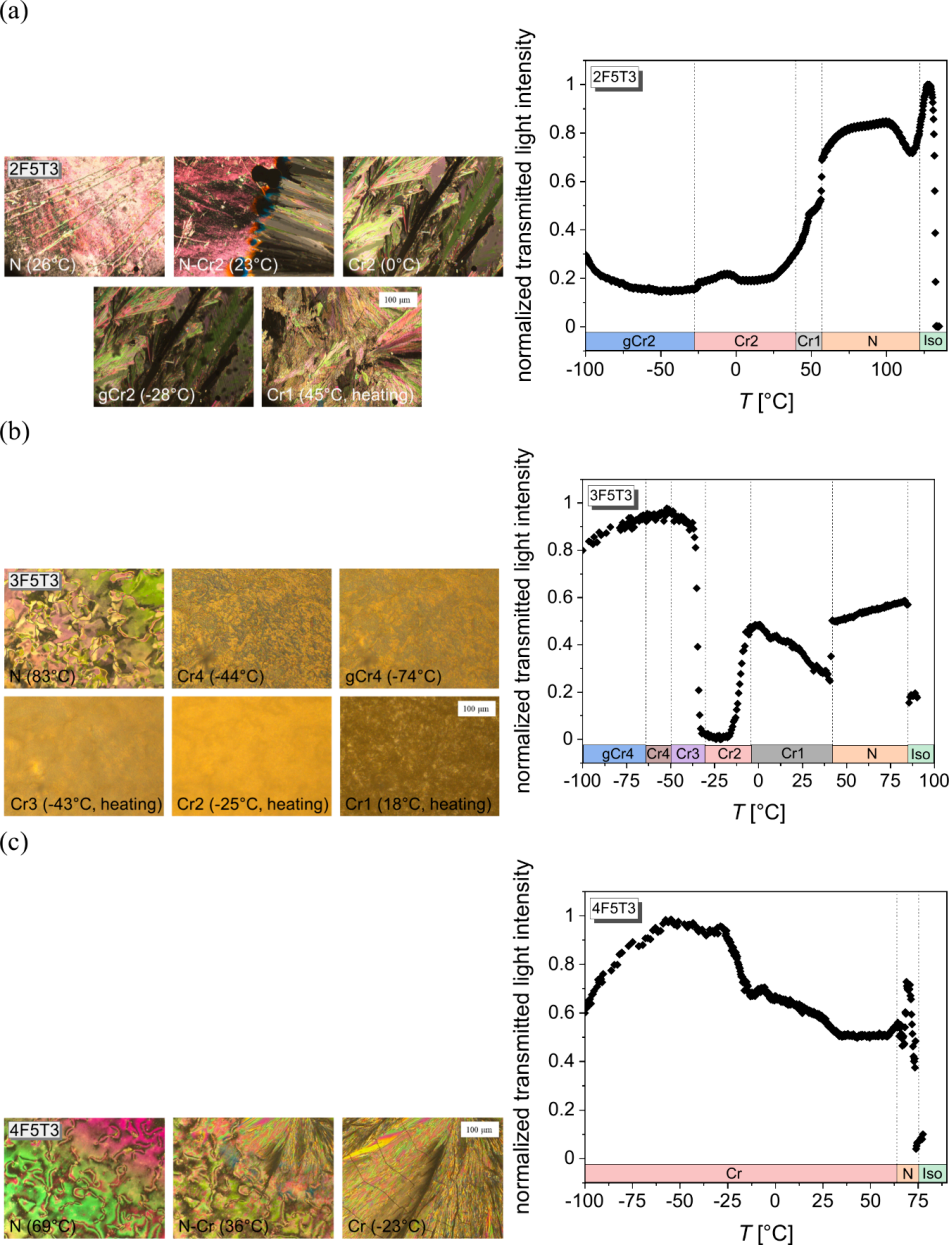
POM textures and TOA analysis of the 2F5T3 (a), 3F5T3 (b), and 4F5T3 (c) registered upon cooling and/or heating at a rate of 5 °C min^–1^. Vertical lines in the TOA plots indicate the transition temperatures obtained by DSC.

Phase transitions in liquid crystals entail the reorganization of molecules, leading to alterations in the anisotropy of the refractive index or birefringence, which can be observed in the distinctive textures captured using the POM technique. The DSC analysis is another method used to identify thermodynamic phases. Anomalies visible on thermograms correspond to specific phase transitions with characteristic values of enthalpy and entropy changes. The DSC thermograms registered upon cooling and/or heating are shown in Figure 3a–c, and the representative POM textures of thermodynamic states of *n*F5T3 compounds with the thermo-optical analysis are presented in Figure 4a–c.

For 2F5T3, the anomaly upon cooling at 122 °C corresponds to the phase transition between the isotropic liquid phase and the nematic phase with the enthalpy change of Δ*H* = 0.9 kJ mol^–1^ and the entropy change of Δ*S* = 2.2 J mol^–1^ K^–1^ (values for the rate of 5 °C min^–1^), Figure 3a. Such small energy is required to rearrange the molecules along the director in the liquid-like N phase. The schlieren texture, characteristic of the N phase, is visible for temperatures above 23 °C; see Figure 4a. At 23 °C, the N-crystal 2 (Cr2) phase transition is registered (with Δ*H* = 12.5 kJ mol^–1^ and Δ*S* = 42.3 J mol^–1^ K^–1^, values for the rate of 5 °C min^–1^). This significant change in the thermal effects indicates the deactivation of the appropriate degrees of freedom. The glass of the Cr2 phase (gCr2 state) is observed below *T*_g_ = −28 °C, with the characteristic kink on thermograms.^32^ Upon heating, after softening of the gCr2 state, the low-temperature Cr2 phase transits into the high-temperature Cr1 phase at 41 °C (with Δ*H* = 13.0 kJ mol^–1^ and Δ*S* = 41.4 J mol^–1^ K^–1^, values for the rate of 5 °C min^–1^). The obtained values of thermal parameters suggest that the Cr2 phase is a more ordered state than the Cr1 phase. The melting of the Cr1 phase occurs at 54 °C (with Δ*H* = 7.1 kJ mol^–1^ and Δ*S* = 2.7 J mol^–1^ K^–1^, values for the rate of 5 °C min^–1^). For lower heating rates, additionally the cold crystallization process is visible at *T*_cc_ = 5 °C. Based on the obtained POM results, the thermo-optical analysis in the TOApy program is applied for each texture, Figure 4a. The transmitted light intensity (TLI) slowly increases upon softening of the gCr2 state. A local maximum of TLI is visible in the Cr2 phase, while in the Cr1 phase the TLI rapidly increases during heating. Two jumps occur on TLI vs temperature dependence, first at 57 °C related to the Cr1–N phase transition and second at 116 °C assigned as the N–Iso phase transition.

For 3F5T3, two anomalies are visible upon cooling, as shown in Figure 3b. One at 83 °C corresponds to the Iso–N phase transition (with Δ*H* = 0.8 kJ mol^–1^ and Δ*S* = 2.2 J mol^–1^ K^–1^, values for the rate of 5 °C min^–1^), and second at −41 °C associated with crystallization to the Cr4 phase (with Δ*H* = 2.4 kJ mol^–1^ and Δ*S* = 10.5 J mol^–1^ K^–1^, values for the rate of 5 °C min^–1^). The irregularities appearing on the thermogram after crystallization are related to continuous ordering of the crystal structure. The glass of the Cr4 phase is observed at *T*_g_ = −67 °C. Both 2F5T3 and 3F5T3 compounds have two flexible terminal alkyl chains; therefore the vitrification of the crystal phase is probably related to the freezing of the conformational disorder. Upon heating, after softening of the glass of 3F5T3, three exothermic processes appear at −51 °C (Cr4–Cr3 transition, with Δ*H* = 2.0 kJ mol^–1^ and Δ*S* = 8.8 J mol^–1^ K^–1^), −33 °C (Cr3–Cr2 transition, with Δ*H* = 0.6 kJ mol^–1^ and Δ*S* = 2.4 J mol^–1^ K^–1^), and −10 °C (Cr2–Cr1 transition, with Δ*H* = 3.4 kJ mol^–1^ and Δ*S* = 12.9 J mol^–1^ K^–1^), values for the rate of 5 °C min^–1^, assigned as the cold crystallizations. These anomalies shift into higher temperatures with increasing heating rates and are characterized by different POM textures, Figure 4b. Also the occurrence of these anomalies only on the nonreversing curve in the MT-DSC regime proves that they are related to cold crystallization processes (inset in Figure 3b). The Cr1–N phase transition occurs at 40 °C (with Δ*H* = 20.8 kJ mol^–1^ and Δ*S* = 66.4 J mol^–1^ K^–1^, values for the rate of 5 °C min^–1^) and is preceded by a pretransitional effect. The TLI slowly increases in the gCr4 state and the Cr4 phase during heating, Figure 4b. This parameter slowly decreases upon Cr4–Cr3 phase transition, while a jump of TLI is observed upon Cr3–Cr2 phase transition. In the Cr2 phase, the TLI has a local maximum, but in the Cr1 phase this parameter decreases with temperature. The melting of the Cr1 phase is associated with the jump of TLI. In the N phase, the TLI slowly increases during heating.

For 4F5T3, the anomaly at 69 °C upon cooling is interpreted as the Iso–N phase transition (with Δ*H* = 0.8 kJ mol^–1^ and Δ*S* = 2.2 J mol^–1^ K^–1^, values for the rate of 5 °C min^–1^), Figure 3c. The next anomaly at 33 °C is related to the crystallization process (with Δ*H* = 19.9 kJ mol^–1^ and Δ*S* = 65.1 J mol^–1^ K^–1^, values for the rate of 5 °C min^–1^), which is also visible on POM texture, Figure 4c. This compound crystallizes upon both fast and slow cooling. The regular cracking detected on the texture of the Cr phase is not a signature of vitrification precisely due to its regularity.^32^ These cracks disappear upon heating, which is visible on the TLI plot as some irregularities, Figure 4c. Upon subsequent heating, the Cr–N phase transition at 65 °C, preceded by a pretransitional effect, and the N–Iso phase transition at 70 °C are visible.

Considering the differences in the number of fluorine atoms in the molecular core of the studied terphenyls, it can be observed that as the number of fluorine atoms increases, the compound transits to the isotropic liquid phase at a lower temperature. Furthermore, the compound with the highest number of fluorine atoms, 4F5T3, does not vitrify, while the remaining derivatives vitrify from the Cr phase. Additionally, the compound with the fewest fluorine atoms, 2F5T3, forms two crystalline phases during heating, whereas the other terphenyls exhibit the presence of a single crystalline phase. As the number of fluorine atoms increases, the temperature range of the nematic phase during heating decreases.

#### 3.1.1 Kinetics of Nonisothermal Cold Crystallization of 3F5T3

**Figure 5:**
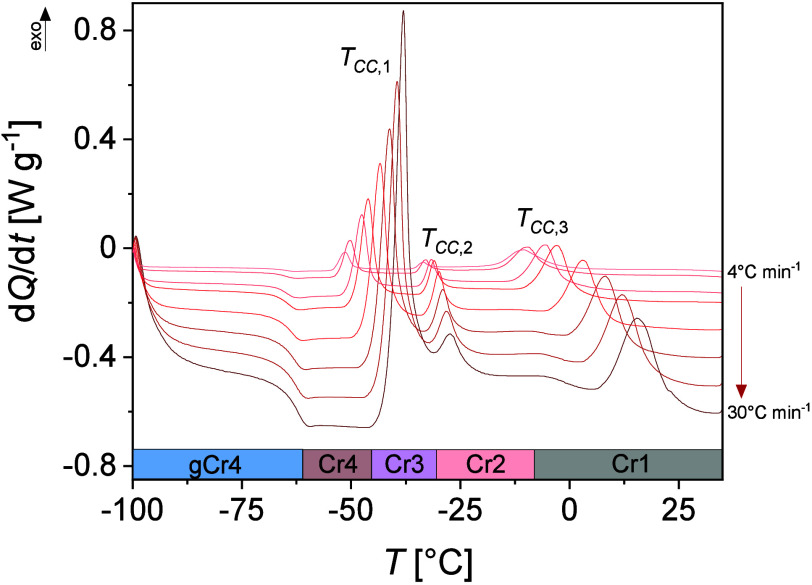
Enlarged area of the cold crystallization processes visible on DSC thermograms registered for various heating rates. *T*_CC,*i*_ (*i* = 1–3) is the cold crystallization temperature.

The cold crystallization process (the crystallization occurring upon heating from the glassy state) may proceed by classical thermodynamic predictions or may occur via diffusion, associated with the mobility of molecules.^33,34^ To describe this process under nonisothermal conditions, the sample is cooled from the isotropic phase to the glassy state, next it is heated, and the DSC thermograms are taken with different heating rates. The cooling rate before the specified heating cycle matched the applied heating rate. For 3F5T3, three cold crystallization processes are observed: the first Cr4–Cr3 process at the cold crystallization *T*_CC,1_ temperature, the second Cr3–Cr2 process at *T*_CC,2_, and the third Cr2–Cr1 process at *T*_CC,3_, Figure 5. The cold crystallization depends on the thermal history of the sample, so the exothermic anomalies related to these processes are shifted toward lower temperatures and their intensities are decreased as the heating rate decreases.

**Figure 6:**
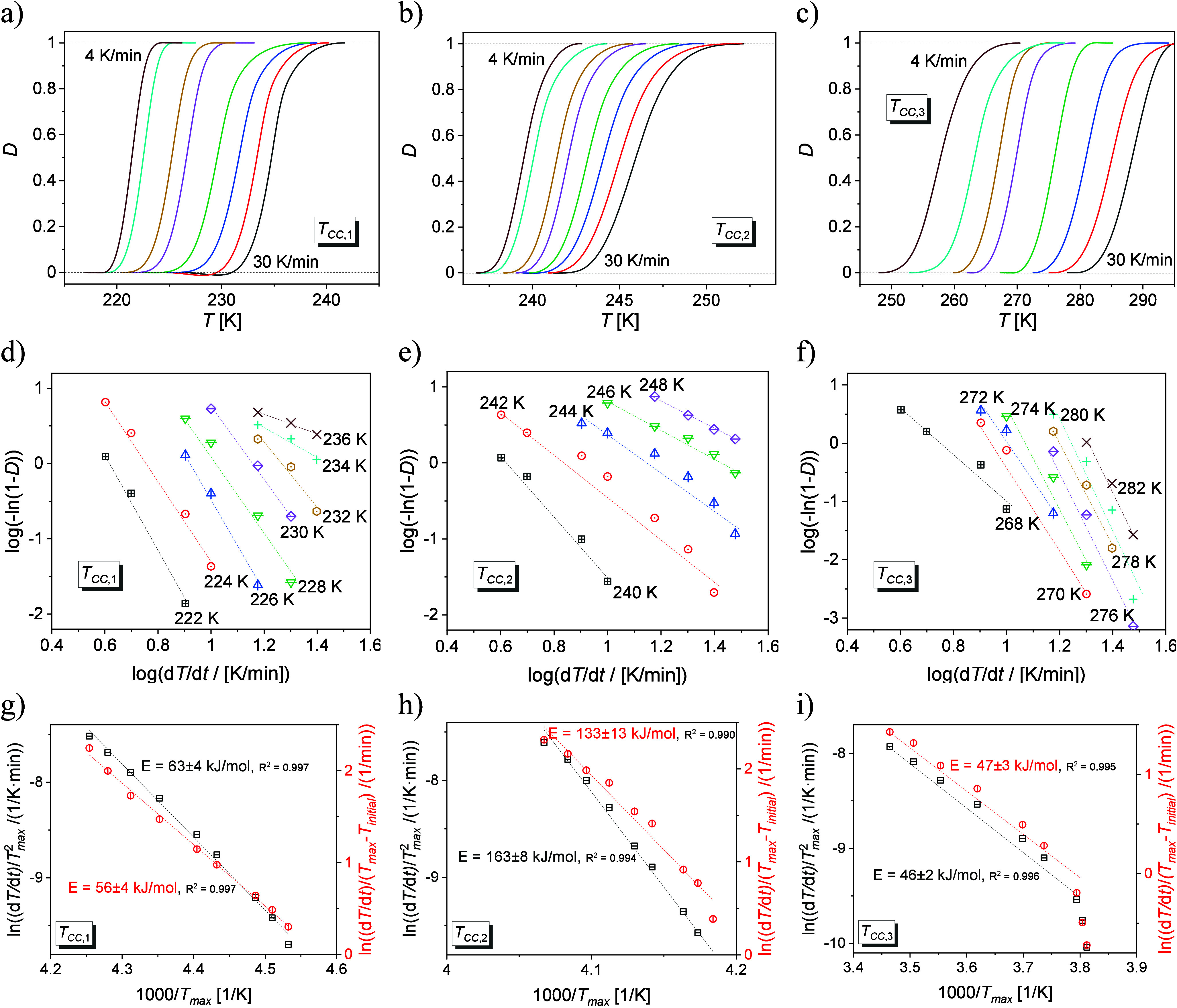
Temperature dependence of the crystallization degree (a–c), Ozawa plots (d–f), Kissinger and Augis–Bennett plots (g–i) for *T*_CC__,1_ (a, d, g), *T*_CC__,2_ (b, e, h), and *T*_CC__,3_ (c, f, i).

The crystallization degree *D* of the nonisothermal process may be calculated for each heating rate d*H/*d*T* according to the formula:^35^
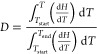
1where *T*_start_ and *T*_end_ are the beginning and ending temperatures of crystallization. Figure 6a–c presents the degree of crystallinity vs temperature determined by DSC thermograms. For all cold crystallization processes, *D* vs *T* curves are shifted toward higher temperatures with increasing heating rate. The knowledge of the *D* allows determination of the kinetics of the nonisothermal cold crystallization processes, using the Ozawa model, preferably in its logarithmic form:^36^
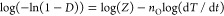
2where *Z* is the Ozawa crystallization rate and *n*_O_ is the Ozawa exponent depending on the crystal size. Ozawa plots display a linear trend, with variations in slope corresponding to the *n*_O_ parameter and changes in the intercept along the vertical axis, represented as log(*Z*), observed for all cold crystallization processes, Figure 6d–f. The values of *n*_O_ and log(*Z*) parameters are presented in Table 1. The *n*_O_ exponent shows a decreasing trend with increasing temperature for the first (the crystal growth dimensionality is reduced from *n*_O_ = 6.59 at 222 K to *n*_O_ = 1.32 at 236 K) and second cold crystallization processes (*n*_O_ is reduced from *n*_O_ = 5.48 at 240 K to *n*_O_ = 1.84 at 248 K), while for the third process the *n*_O_ changes from *n*_O_ = 3.97 at 268 K to *n*_O_ = 10.08 at 282 K. For all cold crystallization processes, the dimensionality of the obtained crystals is different depending on the applied heating rate of the sample. To obtain more information about the mechanism of the cold crystallization processes, the temperature dependence of the log(*Z*) parameter should be discussed. This parameter decreases with increasing temperature for the first and second cold crystallization processes, so the thermodynamic mechanism has an impact on these processes. On the other hand, the log(*Z*) parameter shows the opposite behavior for the third cold crystallization, which means that this process is mainly controlled by diffusion.

**Table 1:** Values of Ozawa *n*_O_ and log(*Z*) Parameters for Three Cold Crystallization Processes

*T*_CC,1_								
*T* [K]	222	224	226	228	230	232	234	236
*n*_O_	6.59	6.40	5.51	5.44	4.72	4.27	2.06	1.32
log(*Z*)	5.93	5.68	5.47	5.39	4.15	4.12	2.96	2.24

*T*_CC,2_					
*T* [K]	240	242	244	246	248
*n*_O_	5.48	3.60	2.41	1.87	1.84
log(*Z*)	3.63	3.14	2.81	2.65	2.06

*T*_CC,3_								
*T* [K]	268	270	272	274	276	278	280	282
*n*_O_	3.97	5.21	6.65	8.30	8.52	8.76	8.92	10.08
log(*Z*)	3.00	5.14	6.69	8.89	9.64	10.81	11.68	12.58

The activation energy of the nonisothermal cold crystallization processes may be determined based on two methods. The Kissinger^37^ formula connects the activation energy (*E*_A_) with the heating rate (d*T*/d*t*) and the maximum temperature (*T*_max_) of the exothermic anomaly via the equation:
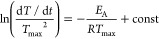
3while the Augis–Bennett^38^ formula includes additionally the initial temperature (*T*_initial_):
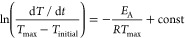
4The linear Kissinger and Augis–Bennett dependences are presented in Figure 6g–i. The values of the activation energies for the first and the third cold crystallization processes equal ca. 60 and 46 kJ/mol, respectively, and are similar for both methods. For the second cold crystallization, the activation energy is 163 (based on the Kissinger formula) and 133 kJ/mol (based on the Augis–Bennett formula). These differences may result from the specificity of the methods used. The Augis–Bennett model is more general, while the Kissinger method was derived for first-order reactions.

**Figure 7:**
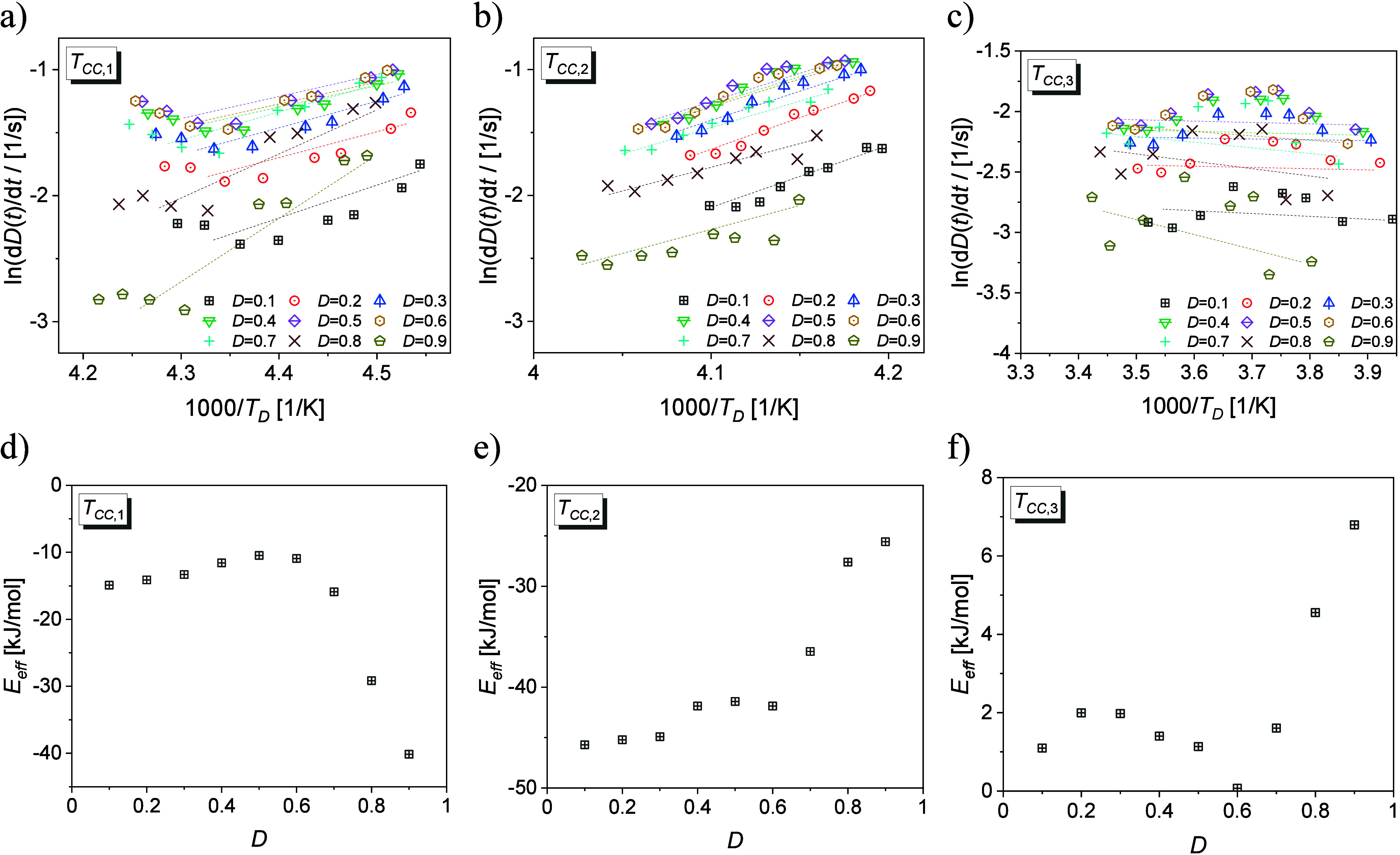
Activation plot based on isoconversional method (a–c) and the effective activation energy vs crystallization degree (d–f) for the *T*_CC,__1_ (a, d), *T*_CC,__2_ (b, e), and *T*_CC,__3_ (c, f).

The data were also analyzed with the isoconversional method which allows determination of the effective activation energy *E*_eff_ as a function of *D* and *T*, according to the formula:^39^
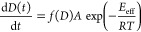
5where *f*(*D*) is the transition model and *A* is a pre-exponential factor. The logarithm of the crystallization rate ln(d*D*(*t*)/d*t*) is plotted against inverted temperature for selected crystallization degree 1000/*T*_D_ (Figure 7a–c) and the value of *E*_eff_ is obtained from the slope of these activation plots. The values of the effective activation energies *E*_eff_ for the first and second cold crystallization processes are negative, meaning that the kinetics of the cold crystallization is controlled by the thermodynamic driving force (Figure 7d,e). The positive value of *E*_eff_ for the third cold crystallization process means the diffusion rate has a bigger impact on the crystallization kinetics (Figure 7f).

### 3.2 Molecular Dynamics of *n*F5T3

**Figure 8:**
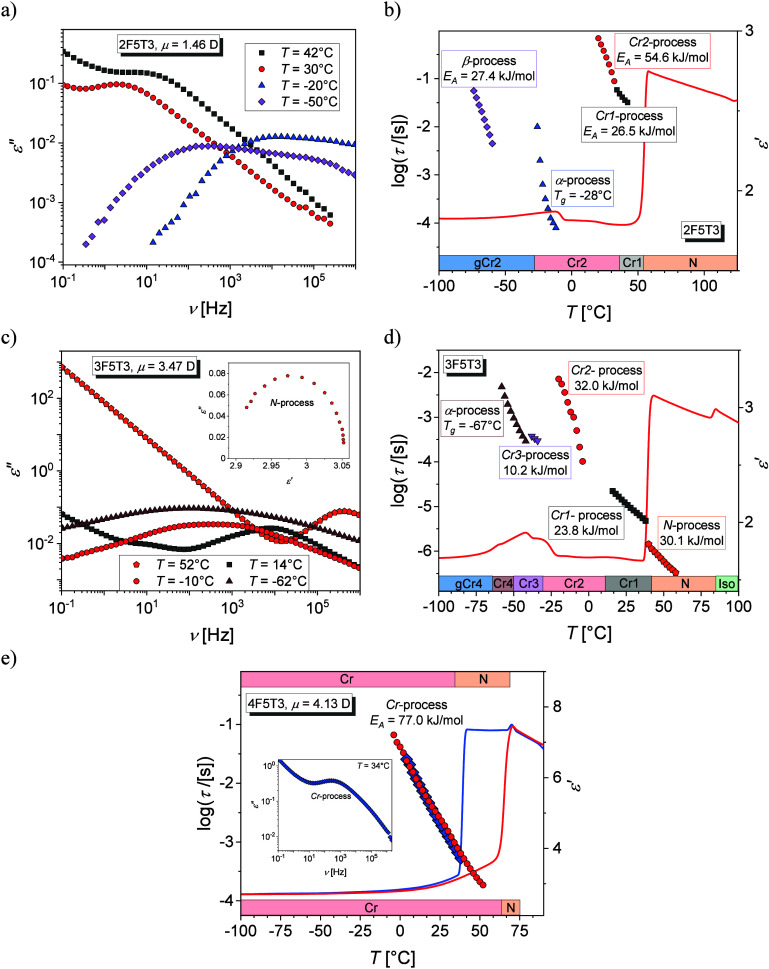
Dielectric loss ε″ vs frequency of relaxation processes at selected temperatures of 2F5T3 (a) and 3F5T3 (c). The temperature dependence of the real part of the dielectric permittivity ε′ measured at 1.33 kHz (lines) and of the relaxation times τ (points) of the relaxation processes of 2F5T3 (b), 3F5T3 (d), and 4F5T3 (e). The inset in panel c presents the Cole–Cole plot for the nematic phase of 3F5T3. The inset in panel e shows the dielectric loss ε″ vs frequency of the Cr-process for 4F5T3.

The relaxation dynamics is described based on BDS results. The individual identified thermodynamic state is characterized by different relaxation processes. The relaxation times of these processes are determined by fitting the experimental complex permittivity in the frequency domain using the Havriliak–Negami (HN) equation:^40^
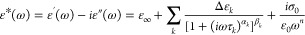
6where ε_∞_ is the permittivity at the high frequency limits, Δε_*k*_ is the dielectric strength of the *k*th process, τ_*k*_ is the dielectric relaxation time of the *k*th dynamic process, σ_0_ is the electric conductivity, ε_0_ is the permittivity of the vacuum, and α_*k*_ and β_*k*_ are shape parameters describing the symmetric and asymmetric broadening of the loss spectra, respectively. For α_*k*_ = 1 and β_*k*_ = 1, the relaxation process is of the Debye type, for 0 < α_*k*_ < 1 and β_*k*_ = 1, it is the Cole–Cole type, and for 0 < α_*k*_ < 1 and 0 < β_*k*_ < 1, it is the HN type. One deals with the ohmic electric conductivity independent of frequency for *n* = 1, and then the quantity σ_0_ is the DC electric conductivity.^41^ For 0 < *n* < 1, one deals with the nonohmic electric conductivity, and then σ_0_ is the quantity proportional to the AC electric conductivity.^42^ The dielectric loss spectra and the temperature dependence of dielectric permittivity at the frequency of 1.33 kHz are shown for 2F5T3 (Figure 8a,b), 3F5T3 (Figure 8c,d), and 4F5T3 (Figure 8e).

In the nematic phase, the relaxation N-processes related to molecular rotations around the short axis (called the low frequency, lf, process) and the long axis (called the high frequency, hf, process) may be observed. Usually, the lf process appears in megahertz frequencies, while the hf process falls in hundred megahertz or gigahertz frequency range. In our BDS experiment, we only observe the lf process in the nematic phase of the 3F5T3 (Figure 8c,d) described by the Cole–Cole model, within the temperature range near the Cr1–N phase transition. In other cases, the relaxation process in the nematic phase occurs outside the measurable frequency range.

**Figure 9:**
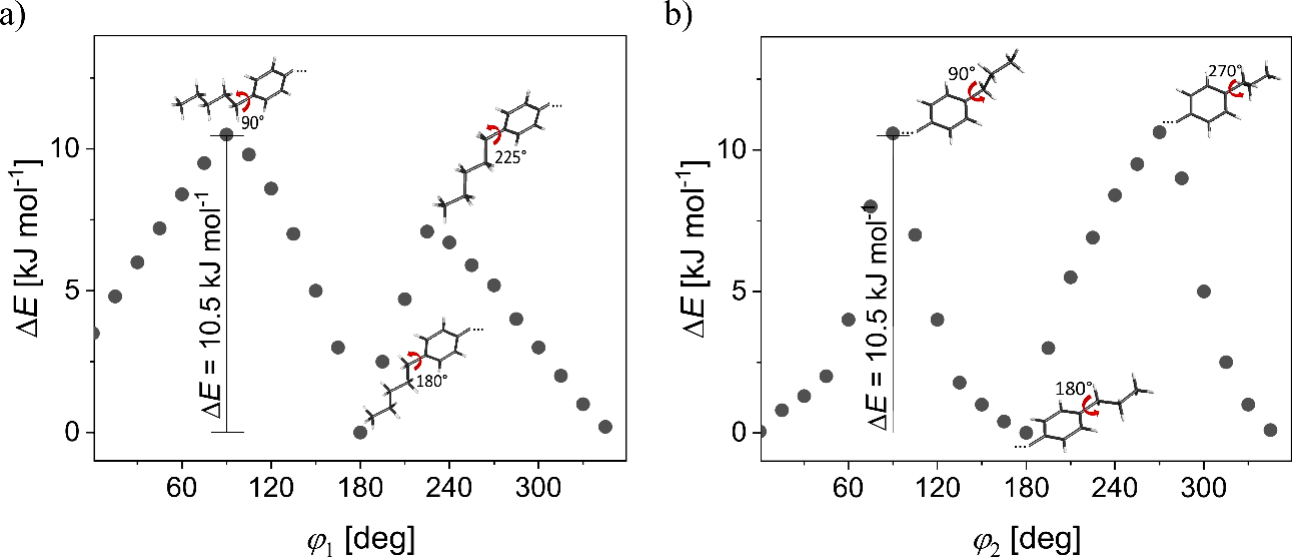
Potential energy surfaces for rotational scans of the φ_1_ torsional angle between the pentyl group and the molecular core (a) and of the φ_2_ torsional angle between the propyl group and the molecular core (b).

The observation of the Cr-processes in the crystal phases for all terphenyls is possible due to the occurrence of fluorine atoms that implicate increasing of the dipole groups.^43^ The relaxation times of the Cr-processes described by the HN model change according to the Arrhenius equation with the activation energies marked in Figure 8b,d,e suggesting that they originate from intermolecular stochastic movements. DFT calculations are carried out to estimate the possible candidates to perform the potential energy scans for changing the torsional angles φ_1_ between the pentyl group and the molecular core (Figure 9a) and φ_2_ between the propyl group and the molecular core (Figure 9b). The energy barriers of rotations of alkyl chains are 10.3 kJ mol^–1^ and 10.5 kJ mol^–1^ for the propyl and pentyl chains, respectively. The two internal dihedrals of the *para*-terphenyl are coupled in same way so the total torsional potential energy is 18 kJ mol^–1^.^44^ According to quantum chemical calculations, we conclude that the values of activation energies of the Cr1-processes for 2F5T3 (Figure 8b) and for 3F5T3 (Figure 8d) correspond to the sum of rotations of the terminal alkyl chains. The activation energies of the Cr2-processes for 2F5T3 (Figure 8b) and for 3F5T3 (Figure 8d) and of the Cr-process for 4F5T3 (Figure 8e) correlate with the changing of torsional angles φ_1_ and φ_2_ coupled with the torsional angles in the molecular core. The Cr3-process of 3F5T3 may be related to the rotation of the propyl chain. The presence of three different relaxation modes in the crystal phases of 3F5T3 may result from the nonsymmetrical position of fluorine atoms in the molecular core in comparison with the remaining terphenyls.

The relaxation modes observed upon heating of the vitrified crystal phases are the secondary β-process described by the Cole–Cole model (visible for 2F5T3) and the structural α-process typical for materials undergoing vitrification fitted with the HN model (visible for 2F5T3 and 3F5T3). The temperature dependence of the α-relaxation times τ_α_ is described by the Vogel–Fulcher–Tammann (VFT) formula:^45^
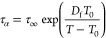
7where τ_∞_ is the pre-exponential factor, *D*_f_ is a constant parameter, and *T*_0_ is the Vogel temperature. The glass transition temperatures *T*_g_ = −28 °C for 2F5T3 and *T*_g_ = −67 °C for 3F5T3, defined as the temperatures at which *τ*_α_ = 100 s, are similar to those determined from the DSC measurements. The relaxation time of the β-process for 2F5T3 changes according to the Arrhenius equation, with the activation energy *E*_A,β_ = 22.4 kJ/mol (Figure 8b). The Ngai and Capaccioli formula allows verification of whether the β-process is the Johari–Goldstein (JG) relaxation:^46^
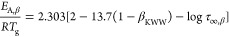
8where β_KWW_ is the stretch parameter of the Kohlrausch–Williams–Watts (KWW) formula^47^ used to fit the α-loss peak (here β_KWW_ = 0.45 for all spectra). Kudlik et al.^48^ proved that *E*_A,β_/(*RT*_g_) = 24 for the β-process classified as the JG mode in many glass formers. For 2F5T3, the equality of eq 8 is not maintained, so the β-relaxation is not of the JG type.

### 3.3 Vibrational Dynamics of 3F5T3

**Figure 10:**
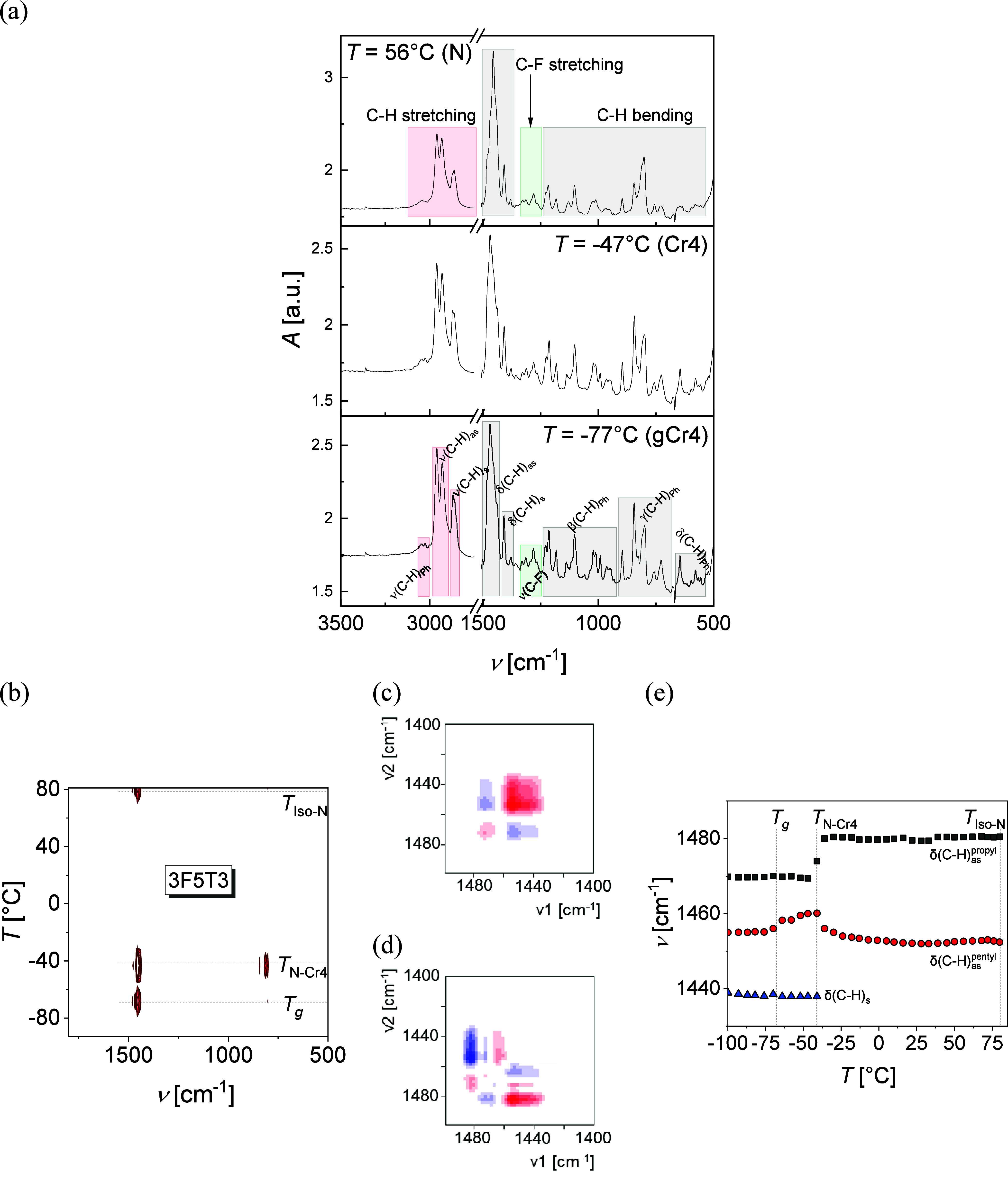
FTIR spectra of the 3F5T3 measured upon fast cooling for selected temperatures at −77 °C in the gCr4 state, at −47 °C in the Cr4 phase, and at 56 °C in the N phase (a). The vibration modes are assigned on the third spectrum: ν-stretching, δ-bending, β-bending in-plane, γ-bending out-of-plane, as-asymmetric, s-symmetric, Ph-in the aromatic ring. Perturbation–correlation moving window two-dimensional (PCMW2D) analysis synchronous map (b) and two-dimensional correlation (2D-COS) analysis synchronous (c) and asynchronous (d) maps. The red and blue areas represent positive or negative correlation intensity, respectively. Temperature dependence of wavenumbers of the δ(C–H) band vibrations (e).

The vibrational dynamics is described for the thermodynamic states of 3F5T3 upon cooling. The absorption infrared spectra consist of many overlapping bands, so the focus is on the description of the bands with the highest intensity, Figure 10a. The first group of bands is C–H stretching vibrations. The bands related to the C–H stretching vibrations in the phenyl rings, ν(C–H)_Ph_, occur above 3000 cm^–1^, while bands originating from C–H asymmetric, ν(C–H)_as_, and symmetric, ν(C–H)_s_, stretching vibrations in the methyl and methylene groups occur below 3000 cm^–1^. The second group of bands is C–F stretching vibrations, ν(C–F), which lie at 1310 cm^–1^. In fluorobenzenes, the C–F groups prefer to form C–H···F interactions instead of F···F.^49^ The third group of bands is C–H bending vibrations. The bands corresponding to the C–H asymmetric, δ(C–H)_as_, and symmetric, δ(C–H)_s_, bending vibrations are located in the range 1500–1300 cm^–1^. Below 1250 cm^–1^ there are absorption bands originating from bending vibrations in the C–H plane in phenyl rings, β(C–H)_Ph_, and below 1000 cm^–1^ from analogous vibrations but outside the plane, γ(C–H)_Ph_. Absorption bands associated with C–H deformation vibrations in phenyl rings, δ(C–H)_Ph_, can be seen below 700 cm^–1^.

To check the influence of temperature on the individual absorption bands, PCMW2D analysis is performed, as shown in Figure 10b. The absorption δ(C–H) bands below 1500 cm^–1^ show positive correlation peaks at temperatures correlated to all phase transitions. The positive correlation peaks are also assigned to γ(C–H)_Ph_ at ca. 800 cm^–1^ during the N–Cr4 phase transition. This suggests that the majority molecular units are involved in the crystallization process, while the mobility of the C–H groups related to bending vibrations is markedly increased in the area of all phase transitions.

To better analyze the behavior of the δ(C–H) bands in the area of 1500–1400 cm^–1^, the synchronous (Figure 10c) and asynchronous (Figure 10d) 2D-COS correlation maps obtained in the temperature region of glass transition are plotted on a plane between two independent wavenumber axes. The negative synchronous cross-peaks for both δ(C–H)_as_ (at 1470 cm^–1^) and δ(C–H)_s_ (at 1440 cm^–1^) indicate different natures of changes of bands intensities. According to Noda’s rules, the positive asynchronous cross-peak at (ν_1_, ν_2_) means that the vibrations related to the ν_1_ band change before that associated with the ν_2_ band.^50^ Considering this, the δ(C–H)_s_ bands change before the δ(C–H)_as_ bands during the glass transition. In the range of 1480–1455 cm^–1^ there are two absorption bands related to δ(C–H)_as_ vibrations in the longer (pentyl part, at 1460 cm^–1^) and shorter (propyl part, at 1480 cm^–1^) alkyl chains of the molecule. The positive sign on the asynchronous cross-peak suggests that the shorter alkyl tail starts ordering before the longer one upon glass transition.

The absorption bands are influenced by both inter- and intramolecular stochastic movements, which can vary not only during phase transitions but also within specific thermodynamic states. For 3F5T3, the δ(C–H) bending vibrations prove an especially sensitive probe of changes to the local surroundings of a molecule.^51^ These bands are distinguished by their significant intensity and are easily analyzed due to their clear separation from other bands. Figure 10e presents changes of wavenumber of the δ(C–H)_s_ band (at 1440 cm^–1^), the δ(C–H)_as_ band at the pentyl chain (at 1460 cm^–1^), and the δ(C–H)_as_ band at the propyl chain (at 1480 cm^–1^) as a function of temperature upon cooling of the sample. In the nematic phase, the position of the band at 1480 cm^–1^ does not change, while the position of the band at 1460 cm^–1^ shifts toward higher wavenumber values as the temperature decreases near the N–Cr4 phase transition. This indicates a weakening of the intermolecular coupling. During crystallization, the band at 1480 cm^–1^ shifts toward lower wavenumber values, in contrast to the band at 1460 cm^–1^, and additionally, the presence of the band at 1440 cm^–1^ becomes apparent. These effects can be explained by the sensitivity of the alkyl chains to the short-range ordering of molecules that occurs first in the crystallization. During the glass transition, there is a sudden change in the position of the band at 1460 cm^–1^, while the positions of the other bands change only slightly. The temperatures at which these dynamic parameter changes occur are in good agreement with phase transition temperatures obtained from POM, DSC, and BDS measurements.

## 4 Conclusions

**Figure 11:**
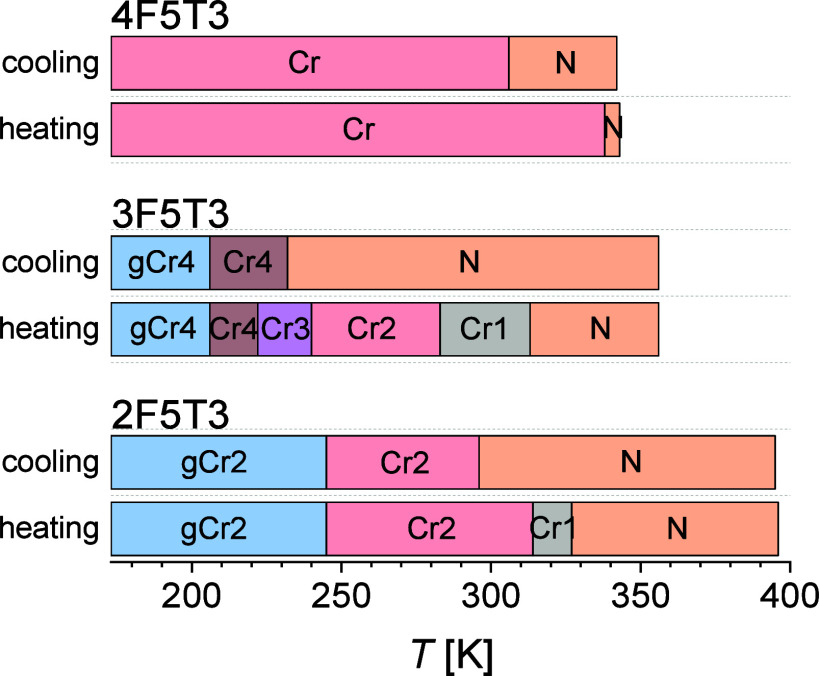
Phase transitions of *n*F5T3 terphenyl derivatives upon cooling and heating were determined from DSC thermograms.

Investigation of three fluorinated 4-pentyl-4″-propyl-1,1′:4′,1″-terphenyls, differing in number and position of fluorine atoms in the mesogenic core, proves that the fluoro-substitution has a significant impact on the self-assembly behavior of these liquid crystals. The plots in Figure 11 present the phase sequence of all terphenyl derivatives upon fast cooling and subsequent heating determined from DSC measurements. As the number of fluorine atoms increases, the transition temperature to the isotropic liquid phase is lower, and the temperature range of the nematic phase upon heating is narrower. Only the 4F5T3 compound with the highest number of fluorine atoms crystallizes, while the other derivatives undergo vitrification from the conformationally disordered crystal phase. Meanwhile, the 2F5T3 compound with the fewest fluorine atoms forms two crystalline phases, while the 4F5T3 terphenyl with the highest fluorine atoms forms only one crystal phase. The 3F5T3 liquid crystal exhibits three cold crystallization processes: the first between Cr4 and Cr3 phases, the second between Cr3 and Cr2 phases, and the third between Cr2 and Cr1 phases. According to the Ozawa and isoconversional methods, the kinetics of the first and the second cold crystallizations depends mainly on the thermodynamic mechanism, while the second cold crystallization is mainly controlled by diffusion.

For all analyzed terphenyls in the crystal phases, relaxation processes are observed, differing in activation energy and thus in the type of intermolecular stochastic movements responsible for these processes. For instance, the Cr-processes may correspond with the rotation of the alkyl chain or with the sum of rotations of both terminal alkyl chains with/without the torsional angles in the molecular core. The cause may be the different arrangement of fluorine atoms in the rigid core, which can result in a varied distribution of the most energetically favorable molecular conformations as a function of temperature. The infrared absorption spectra of the 3F5T3 compound with the nonsymmetrical position of fluorine atoms in the mesogenic core prove that especially the bending vibrations of C–H groups in the pentyl and propyl chains are the most sensitive to structural changes occurring during phase transitions.
